# MicroRNA-10b regulates epithelial–mesenchymal transition by modulating KLF4/KLF11/Smads in hepatocellular carcinoma

**DOI:** 10.1186/s12935-018-0508-0

**Published:** 2018-01-17

**Authors:** Gulibaha Hujie, Sheng-hua Zhou, Hua Zhang, Jie Qu, Xiao-wei Xiong, Outikuer Hujie, Cheng-gong Liao, Shun-e Yang

**Affiliations:** 1 Department of Medical Oncology, Affiliated Tumor Hospital of Xinjiang Medical University, Urumqi, 830000 People’s Republic of China; 2 Department of Gerontology, General Hospital of Xinjiang Military Command of PLA, Urumqi, 830000 People’s Republic of China; 3 Department of Oncology, Korla Hospital, Second Divisions of Xinjiang Production and Construction Corps, Korla, 841000 People’s Republic of China; 4 Xinjiang Medical University, Urumqi, 830000 People’s Republic of China; 5 Department of Oncology, General Hospital of Xinjiang Military Command of PLA, Urumqi, 830000 People’s Republic of China

**Keywords:** miR-10b, Hepatocellular carcinoma, Epithelial–mesenchymal transition, KLF4, KLF11

## Abstract

**Background:**

Our previous work showed that miR-10b was overexpressed in hepatocellular carcinoma (HCC) and promoted HCC cell migration and invasion. Epithelial–mesenchymal transition (EMT) is involved in HCC metastasis. So, we suspected that miR-10b might participate in the HCC EMT.

**Methods:**

We performed morphological analysis and immunofluorescence to observe the roles of miR-10b in HCC EMT. The expression of KLF11 and EMT markers were detected by real-time RT-PCR and western blot. The regulation roles of miR-10b on KLF11 and KLF4 were determined by luciferase reporter assay. The chromatin immunoprecipitation revealed the binding relationship between KLF4 and KLF11.

**Results:**

We found that overexpression of miR-10b could promote HCC EMT. miR-10b could upregulated KLF11 expression. The upregulation of KLF11 reduced the downstream molecular Smad7 expression, which upregulated the Smad3 expression to promote EMT development. Furthermore, the induction role of miR-10b in HCC EMT could be blocked by KLF11 siRNA. But our results showed that there was no direct regulation of miR-10b in KLF11 expression. Specifically, miR-10b could bind to the 3′UTR of KLF4 and inhibit KLF4 expression. KLF4 could directly bind to KLF11 promoter and downregulate KLF11 transcription.

**Conclusion:**

Our results reveal that miR-10b downregulates KLF4, the inhibitory transcriptional factor of KLF11, which induces Smads signaling activity to promote HCC EMT. Our study presents the regulation mechanism of miR-10b in EMT through the KLF4/KLF11/Smads pathway for the first time and implicates miR-10b as a potential target for HCC therapies.

## Background

Hepatocellular carcinoma (HCC), the most common primary malignant liver cancer, is a global public health problem that accounts for approximately 500,000 deaths annually [1]. The high recurrence and low 5-year survival rate of HCC are mainly due to the intrahepatic and extrahepatic metastases [2], and the rate of recurrence is 86.5% for intrahepatic metastasis and 13.5% for extrahepatic metastasis [3]. The epithelial–mesenchymal transition (EMT) plays a pivotal role in local invasion and distant metastasis during HCC progression [4]. However, the mechanism underlying the EMT of HCC is largely unknown.

Current studies have shown that microRNAs (miRNAs) could act as activators or inhibitors of tumor metastasis by targeting multiple signaling pathways involved in metastasis [5]. Moreover, miRNAs have been implicated in the process of EMT through the modulation of EMT-related genes [6]. Our previous results suggested that miR-10b was overexpressed in HCC and promoted HCC cell migration and invasion through the HOXD10/RhoC/uPAR/MMPs pathway [7]. MMPs help cancer cells spread by breaking down the extracellular matrix (ECM) and other barriers which play an important role in the EMT progress [8]. So, we speculate that miR-10b may involve in EMT development.

Krüppel-like factor 11 (KLF11) belongs to the family of Sp1/Krüppel-like transcription factors and has been initially characterized as a TGF-β inducible early gene in the EMT progress [9]. KLF11 promotes the EMT through binding to the Smad7 promoter and suppressing the transcription of Smad7, which interrupts the Smad7-driven negative feedback loop [10]. Additionally, KLF11 can directly upregulate the Smad2/3 expression to promote EMT development [11]. In this study, we determined whether miR-10b was involved in KLF11 regulation and whether it participated in HCC EMT progress.

In this study, we first found that miR-10b could promote EMT in HCC cells. Then, we identified the regulation mechanism of miR-10b in EMT through the KLF4/KLF11/Smads pathway. Thus, our data suggested important roles for miR-10b in HCC EMT and implicated miR-10b as a potential target for HCC therapies.

## Methods

### Cell lines and culture conditions

The two HCC cell lines were used in this study: MHCC-97H (HCC cells with high metastatic potential) and MHCC-97L (HCC cells with low metastatic potential) [12]. All cell lines were purchased from Shanghai Institute for Biological Sciences (Shanghai, China). All cell lines were routinely cultured in RPMI-1640 medium (Hyclone Laboratories, Logan, UT) supplemented with 10% fetal calf serum (Gibco BRL, Rockville, MD, USA) at 37 °C in a humidified atmosphere of 5% CO_2_.

### Immunofluorescence

Cells were seeded in 4-well 35-mm dishes (Greiner Bio-One North America Inc., Monroe, NC, USA) at a density of 1000 cells/well and grown for 48 h in culture medium. Then cells were fixed in 4% paraformaldehyde for 20 min and permeabilized in phosphate-buffered saline (PBS) supplemented with 0.5% Triton X-100. After blocking, cells were incubated with the indicated antibodies for 2 h. Cells were washed in PBS, incubated with their corresponding FITC-labeled or TRITC-labeled secondary antibodies (Pierce, Rockford, IL, USA) for 1 h at room temperature and stained with DAPI (Vector Labs, Burlingame, CA, USA). Finally, the cells were mounted using glycerol and observed using a Nikon A1 laser scanning confocal microscope (Japan).

### Western blot

Cell samples were lysed with RIPA buffer (Beyotime, China). Equal amounts (10 μg) of total protein were loaded, and then subsequently immunoblotted with the primary antibodies, including anti E-cadherin (BD Biosciences, Franklin Lakes, USA), Vimentin (Invitrogen, Carlsbad, CA, USA), KLF4, KLF11, Smad7, Smad3 and tubulin (Santa Cruz, CA, USA). Proteins were detected using the Amersham enhanced chemiluminescence system (Pierce, Rockford, IL, USA) according to the manufacturer’s instructions.

### Real-time RT-PCR

Real-time RT-PCR was performed as described previously [13]. Expression data were uniformly normalized to glyceraldehyde-3-phosphate dehydrogenase (GAPDH) as an internal control, and the relative expression levels were evaluated using the *ΔΔ*Ct method [14, 15]. Primers were used as described previously [16, 17].

### Vector construction, siRNA, and luciferase reporter assay

The miR-10b expression plasmid was constructed by our laboratory previously [7]. Cultured cells were transfected with miR-10b expression vector, antisense miR-10b (anti-miR-10b), scramble miRNA using Lipofectamine 2000 (Invitrogen) according to the manufacturer’s protocol. The sequence were described as before [7, 18].

The 3′UTR segment of KLF4 and KLF11 were subcloned into the pmirGLO vector (Promega, Madison, WI, USA), respectively [11, 19]. The coding sequences of KLF4 was amplified and cloned into pcDNA3.1 (Invitrogen) [20]. The promoter of the KLF11 gene (about − 2000 upstream to the Exon1, containing the KLF4 binding sites CACCC) was amplified by PCR and inserted into the pGL3-basic vector (Promega). The mutant constructs were generated using a QuickChange mutagenesis kit (Stratagene, La Jolla, CA, USA). All constructs were further confirmed by sequencing. siRNAs targeting KLF4, KLF11 and negative control siRNA were purchased from Ambion. Cell transfection and dual luciferase reporter assay were performed as described previously.

### Chromatin immunoprecipitation (ChIP)

ChIP assays were performed using a EZ ChIP assay kit (Millipore Corporation, Billerica, MA, USA). Immunoprecipitation was carried out with anti-KLF4 antibody or rabbit IgG at 4 °C overnight with rotation. The immunoprecipitated DNA was amplified by the promoter-specific primers: forward 5′-ACG CTG AGT ACA GTG GGA GCC AC-3′; reverse 5′-TCC TCG AGC CTG CAT T-3′. The PCR products were analyzed on 1% agarose gel.

### Statistical analysis

All statistical analyses were performed using the SPSS statistical software package (SPSS, Chicago, IL, USA). The significance of the data was determined using Student’s *t* test or one-way ANOVA. All the statistical tests were two-sided, and a *P* value < 0.05 was considered significant.

## Results

### miR-10b promotes EMT in HCC cells

**Fig. 1:**
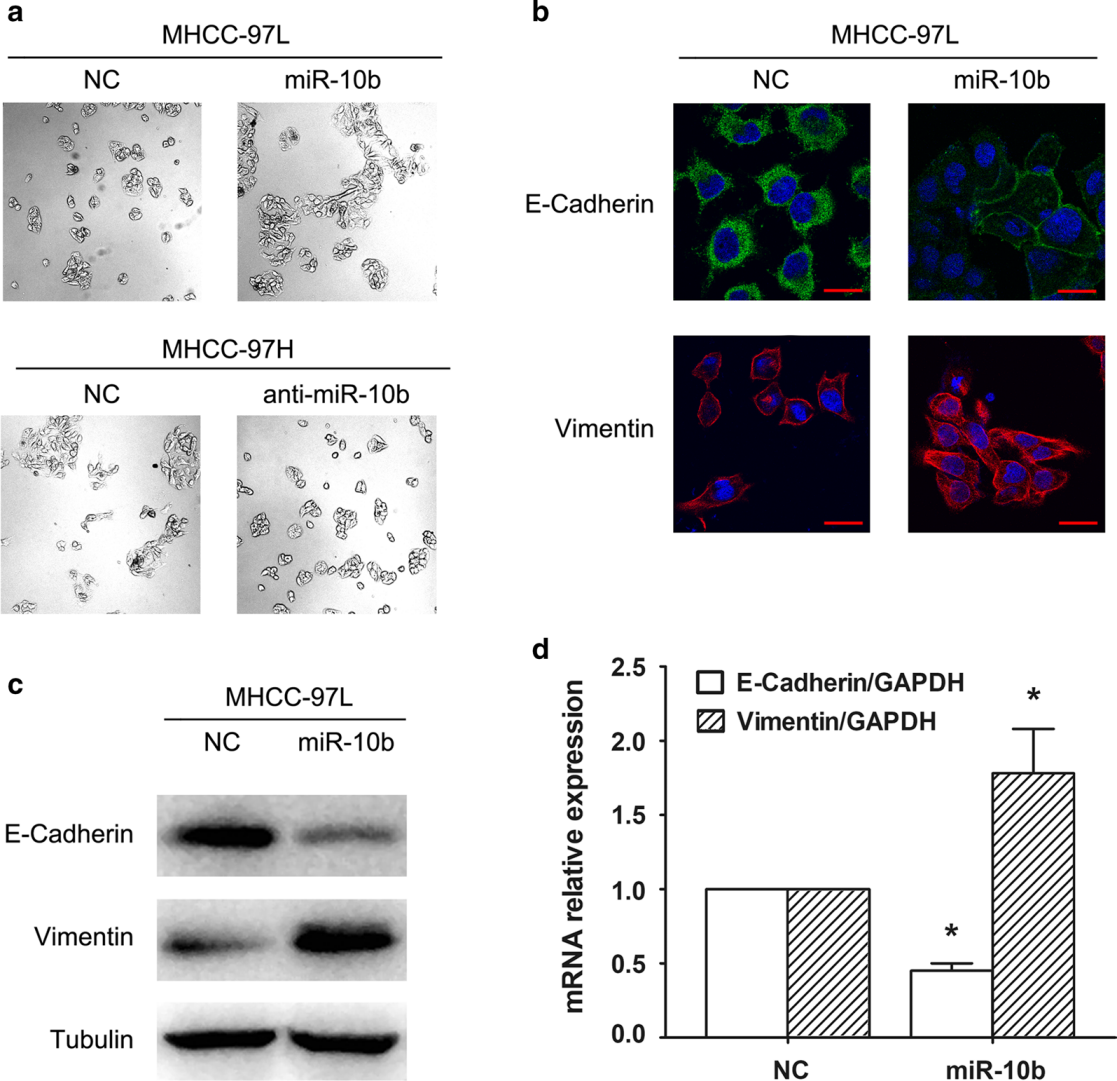
miR-10b promotes EMT in HCC cells. **a** In MHCC-97L cells transfected with miR-10b overexpression plasmid and in MHCC-97H cells transfected with anti-miR-10b, morphological changes were observed under a light microscope. **b** Expression of E-cadherin and Vimentin in MHCC-97L cells with miR-10b overexpression were examined by immunofluorescence. Fluorescence was observed by confocal laser-scanning microscopy. Scale bars, 20 μm. **c**, **d** The E-cadherin, Vimentin protein and mRNA levels were detected by western blot and real-time RT-PCR after overexpression of miR-10b in MHCC-97L cells. *P < 0.05

Morphological analysis showed that MHCC-97L cells transfected with miR-10b expression plasmid exhibited a greater number of mesenchymal cells. The MHCC-97L cells lost their cobblestone pattern and acquired a spindle-shaped morphology. As shown in Fig. 1a, the cells showed a spindle-shaped, fibroblast-like morphology. In contrast, inhibition of miR-10b expression reversed the EMT phenotype of MHCC-97H cells (Fig. 1a). Then, the epithelial and mesenchymal markers were detected. We performed an immunofluorescence using E-cadherin and vimentin as epithelial and mesenchymal markers, respectively. As shown in Fig. 1b, the expression of E-cadherin was downregulated, and the mesenchymal marker vimentin was upregulated after transfection of miR-10b compared with that of control. Furthermore, we observed a decrease of E-cadherin at both the protein and mRNA levels transfected with miR-10b in response to negative control (Fig. 1c, d). On the contrary, vimentin was increased in protein and mRNA (Fig. 1c, d). Altogether, these results demonstrated that miR-10b played an important role in the regulation of EMT in HCC cells.

### miR-10b induces upregulation of KLF11 and Smads signaling activity to promote EMT

**Fig. 2:**
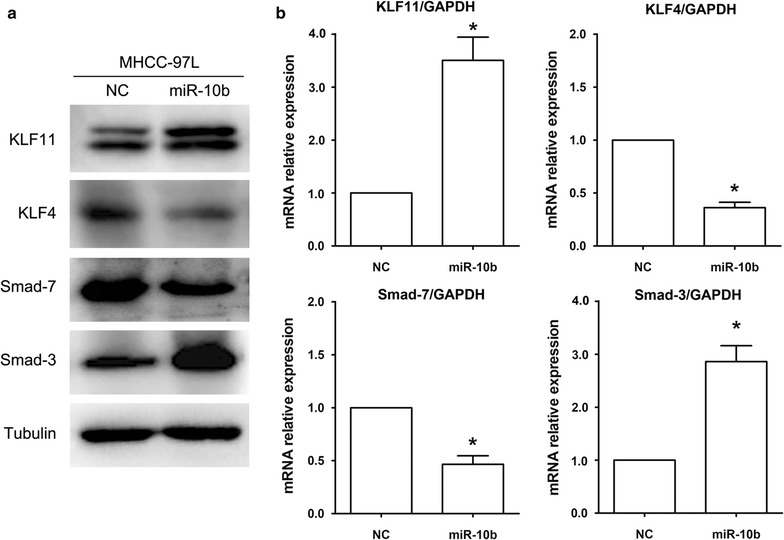
miR-10b regulates KLF11, Smad7 and Smad3 expression in HCC cells. **a** The protein expression levels of KLF11, KLF4, Smad7 and Smad3 were measured in MHCC-97L cells transfected with miR-10b plasmid by western blot. **b** mRNA expression levels of KLF11, KLF4, Smad7 and Smad3 as above were detected by real-time RT-PCR. *P < 0.05

Because previous studies have shown that KLF11 potentiates TGF-β/Smads signaling activity by suppressing the expression of Smad7, we explored whether miR-10b affects EMT progress by modulating the expression of KLF11 and Smad7. As shown in Fig. 2a, b, KLF11 protein and mRNA expression levels increased after transfected with miR-10b. Consequently, the Smad7 expression was decreased. Since the inhibition of Smad7 was reduced, the Smad3 protein and mRNA expression levels were increased. Our results indicated that the levels of KLF11 were increased by miR-10b, which induced Smads signaling activity to promote EMT.

### KLF4 but not KLF11 is a direct target of miR-10b

**Fig. 3:**
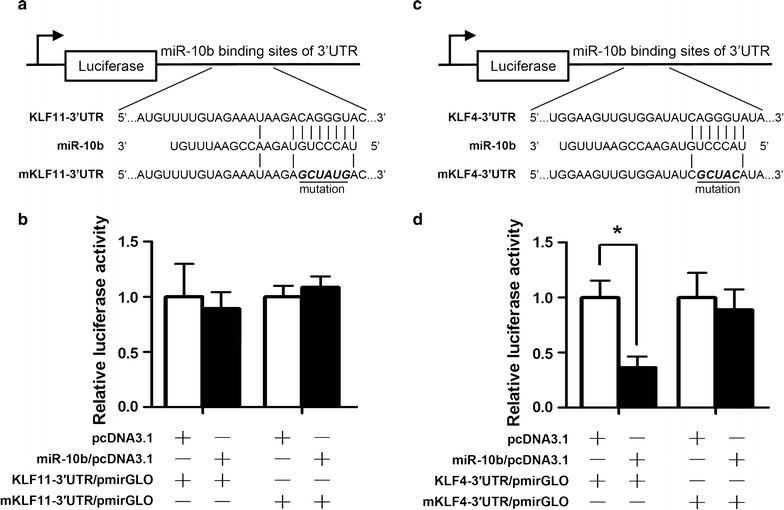
The KLF4 3′UTR is a target of miR-10b. **a**, **c** Upper panel, predicted duplex formation between miR-10b and 3′UTR of KLF11 or KLF4. Lower panel, diagram of the luciferase reporter plasmids: plasmid with the full length KLF11 and KLF4 3′UTR insert and plasmid with a mutant KLF11 and KLF4 3′UTR which carried a substitution of five or six nucleotides within the miR-10b binding site, respectively. **b**, **d** The relative luciferase activity in MHCC-97L cells were determined after the KLF11 and KLF4 3′UTR or mutant plasmids were co-transfected with miR-10b overexpression vector or negative control, respectively. *P < 0.05

Using the target prediction analysis, we found that a conserved sequence in the 3′UTR of KLF11 mRNA has a perfect match to the seed sequence of miR-10b. To verify whether miR-10b directly targeted KLF11 in HCC cell lines, luciferase reporter assays were carried out. We constructed KLF11-3′UTR/pmirGLO and mKLF11-3′UTR /pmirGLO with the miR-10b binding site (Fig. 3a). Cotransfection of MHCC-97L cells with KLF11-3′UTR/pmirGLO and miR-10b/pcDNA3.1 caused no decrease in the luciferase activity compared with the negative control (*P* > 0.05, Fig. 3b). The similar result was found in transfection with mutation plasmid (*P* > 0.05, Fig. 3b). So, we found that there was no direct regulation of miR-10b in KLF11 expression. Our results showed that overexpression of miR-10b could significantly increase the KLF11 expression. We speculated that there was a regulation mediator factor between miR-10b and KLF11.

KLF4 has been reported to be regulated by miR-10b in gastric carcinoma [19]. To verify whether miR-10b directly targeted KLF4 in HCC cell lines, we constructed the KLF4 3′UTR luciferase reporter plasmid (Fig. 3c). There was a 63% decrease in the luciferase activity after co-transfection of KLF4-3′UTR/pmirGLO and miR-10b/pcDNA3.1 compared with the negative control (*P* < 0.05, Fig. 3d). This suppression was rescued by the five-nucleotide substitution in the core binding sites. All these results indicated that miR-10b exerts inhibitory effects on KLF4 expression via interaction with the 3′UTR of KLF4.

### KLF4 directly binds to the KLF11 promoters and negatively regulates its expression

**Fig. 4:**
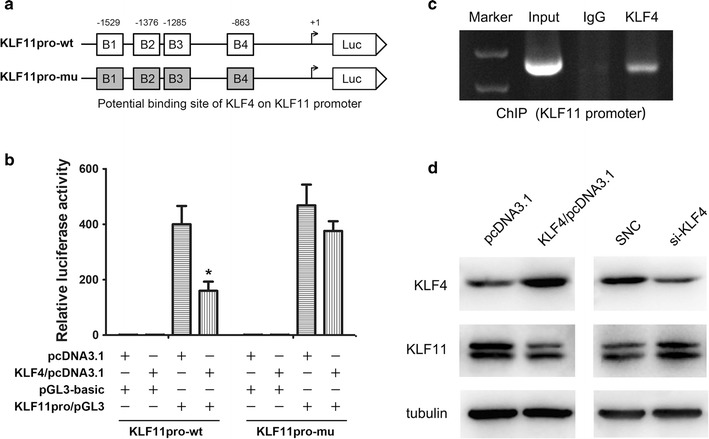
KLF4 regulates KLF11 expression in HCC cells. **a** Dual-luciferase reporter assay was performed by cotransfection of the KLF11 promoter wildtype fragment or mutated fragment with overexpression of KLF4. **b** Reporter assay in HCC cells transfected with KLF11 promoter constructs with mutations in potential binding elements for KLF4. wt, wild type; mu, mutation type. Luciferase activity was expressed as relative to that of the pGL3-basic vector (a promoter-less vector). *P < 0.05, by one-way ANOVA. **c** ChIP assay demonstrated KLF4 binding to the KLF11 gene promoter. **d** KLF4 and KLF11 protein expression in cells transfected with KLF4 overexpression vector or siRNAs

To determine the role of KLF4 in KLF11 transcription, we cloned the human KLF11 promoter fragment into the pGL3 luciferase vector for a luciferase activity assay. The transcriptional activity of KLF11 was reduced by KLF4 overexpression (Fig. 4a, b). These results suggested that KLF4 participated in the regulation of KLF11 transcriptional activity. To validate this notion, we mutated these binding sites individually and used them in a reporter assay. The results showed that the mutations in KLF4 binding sites in the KLF11 promoter significantly impaired the effect of KLF4 on KLF11 transcription activation (Fig. 4b), suggesting that KLF4 can bind to its special binding motifs on KLF11 promoter to down-regulate their transcription.

Furthermore, we performed in vivo ChIP assays to investigate whether KLF4 binds to KLF11 promoter regions. The ChIP assays revealed that endogenous KLF4 directly bound to KLF11 promoters (Fig. 4c). To further assess the biological roles of KLF4 in KLF11 expression, we applied loss- and gain-of-function approaches. Western-blot showed that KLF11 protein expression was downregulated or upregulated after overexpression or siRNA knockdown of KLF4, respectively (Fig. 4d). Together, these results suggested that KLF4 served as a transcription factor that inactivated KLF11 transcription and down-regulated their expression. So, we concluded that miR-10b downregulated KLF4, the inhibitory transcriptional factor of KLF11, which induced Smads signaling activity to promote HCC EMT.

### miR-10b promotes EMT in HCC cells via upregulation of KLF11

**Fig. 5:**
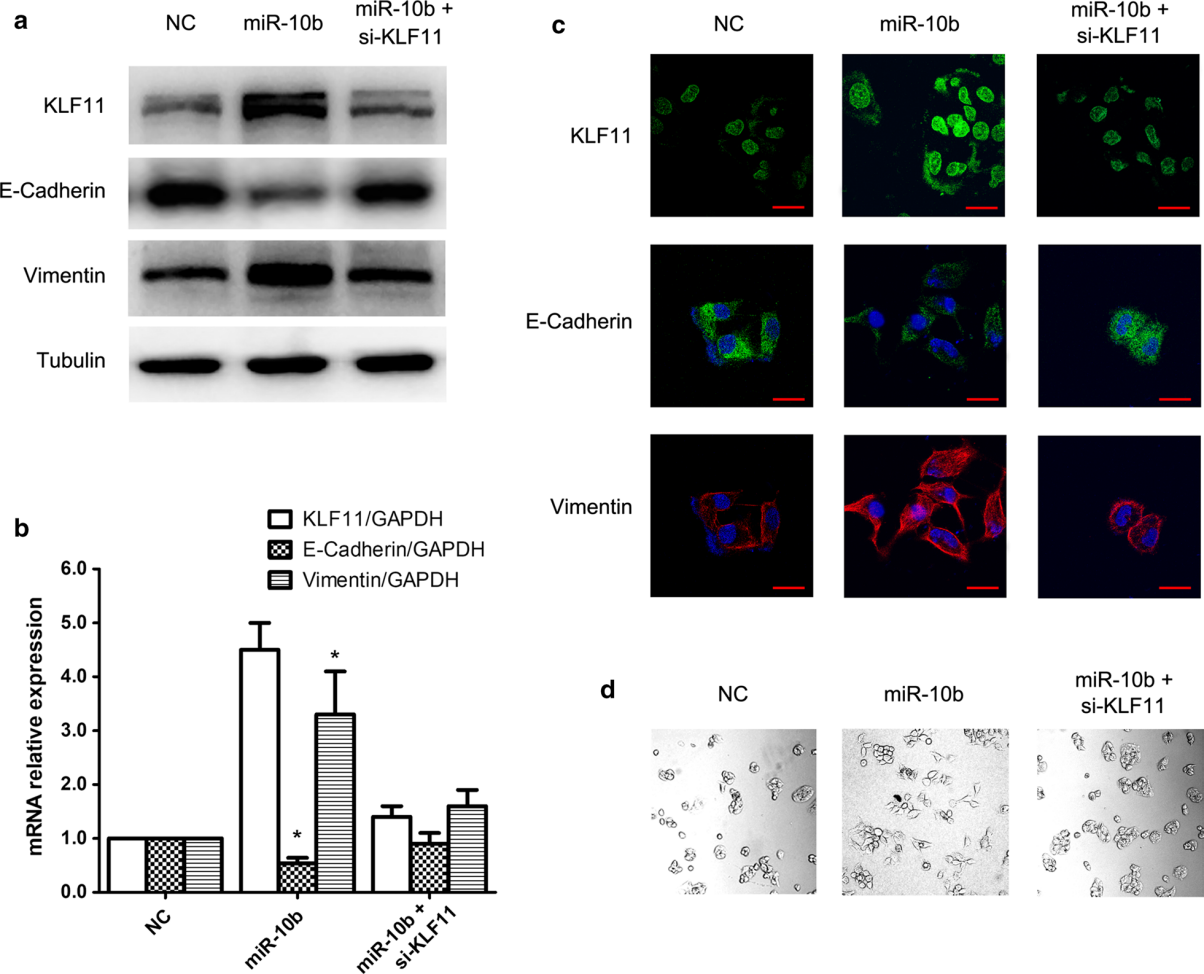
miR-10b promotes EMT in HCC cells via upregulation of KLF11. **a** KLF11, E-cadherin and Vimentin expression in HCC cells treated with miR-10b or rescued with si-KLF11 by western blotting. **b** Real-time RT-PCR analyses of KLF11, E-cadherin and Vimentin mRNA levels as above were transfected. **c** The expression levels of KLF11, E-cadherin and Vimentin were examined in HCC cells treated with miR-10b or si-KLF11 by immunofluorescence. Fluorescence was observed by confocal laser-scanning microscopy. **d** Morphological changes were observed under a light microscope. *P < 0.05

**Fig. 6:**
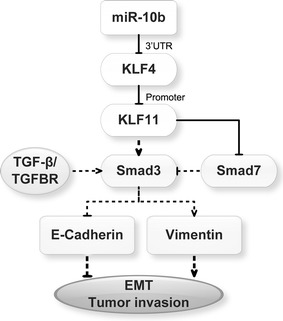
A schematic representation of the major molecular mechanism of miR-10b promotes EMT through KLF4/KLF11/Smads pathway. (The signal pathways showed as dotted line have been previously demonstrated)

To confirm the role of miR-10b in HCC EMT via its regulation on KLF11 expression, we downregulated KLF11 expression through transfecting siRNA to block miR-10b regulation. As expected, transfection of the miR-10b expression plasmid resulted in increased KLF11 expression. By contrast, si-KLF11 transfection eliminated the upregulation induced by miR-10b. The EMT markers E-cadherin and vimentin were also detected. Our results showed that the induction of miR-10b was blocked by KLF11 siRNA (Fig. 5a). The realtime-RT PCR also confirmed the western blot results (Fig. 5b). The immunofluorescence assay also displayed the same change (Fig. 5c). We further observed the morphological change. Our results showed that the HCC cells exhibited a greater number of mesenchymal cells transfected with miR-10b expression plasmid, which was restored by si-KLF11 (Fig. 5d). Our results indicated that miR-10b significantly induced HCC EMT by up-regulating KLF11 expression. Altogether, we identified the regulation mechanism of miR-10b in EMT through the KLF4/KLF11/Smads pathway. A summary diagram that outlines the above-described regulatory network is shown in Fig. 6.

## Discussion

In our study, we found that overexpression of miR-10b could promote HCC EMT. miR-10b induces upregulation of KLF11 and Smads signaling activity to promote EMT. Specifically, there was no direct regulation of miR-10b in KLF11 expression. Our results showed that miR-10b downregulates KLF4, the inhibitory transcriptional factor of KLF11 to upregulate KLF11 expression. Our study presents the regulation mechanism of miR-10b in EMT through the KLF4/KLF11/Smads pathway for the first time.

HCC is the third leading cause of cancer-related death worldwide. Since clinical symptoms are not easily observed during the early stage, the prognosis is poor at the time of diagnosis, which, in most cases, is during the advanced stage [21]. Thus, it is of much significance to explore new diagnostic and therapeutic molecular targets for HCC. miRNAs have been demonstrated to have close relationship with HCC. miR-10b locates in the HOX gene cluster on chromosome 2, suggesting that it is closely related to tumor invasion and metastasis. Previous studies showed that miR-10b was overexpressed in a variety of human cancers, such as breast cancer, malignant glioma, nasopharyngeal carcinoma, pancreatic cancer, and HCC [22].

EMT is a key process driving cancer metastasis and the loss of E-cadherin and increase in vimentin expression are considered to be the most important molecular markers of EMT. Recent studies have revealed that miRNAs act as crucial modulators of EMT through the regulation of E-cadherin and other molecules such as vimentin and ZEB [23]. Our previous work has demonstrated that miR-10b was overexpressed in HCC and promoted HCC cell migration and invasion [7]. In this study, we found that transfected with miR-10b expression plasmid exhibited a greater number of mesenchymal cells. The expression of E-cadherin was downregulated, and the mesenchymal marker vimentin was upregulated after transfection of miR-10b compared with that of control. These findings demonstrated that miR-10b promoted EMT in HCC cells.

Next, we explored the underlying mechanisms involved in the regulation of EMT by miR-10b. We found that KLF11 protein and mRNA expression levels increased after transfected with miR-10b. Consequently, the Smad7 expression was decreased. Subsequently, the Smad3 protein and mRNA expression levels were increased. So, our results indicated that the levels of KLF11 were increased by miR-10b, which induced Smads signaling activity to promote EMT. We speculated that miR-10b could bind to the KLF11 3′UTR and regulate its expression. We constructed KLF11-3′UTR luciferase reporter plasmid. Cotransfection of KLF11-3′UTR and miR-10b caused no decrease in the luciferase activity compared with the negative control. The similar result was found in transfection with mutation plasmid. These results indicated that there was no direct regulation of miR-10b in KLF11 expression. Thus, we speculated that there was a regulation mediator factor between miR-10b and KLF11.

Bioinformatic prediction tools indicated that KLF4 is a putative target of miR-10b [24]. Zhang has identified that miR-10b targeted KLF4 levels in human NPC cells [25]. Consistently, we found that miR-10b exerts inhibitory effects on KLF4 expression via interaction with the 3′UTR of KLF4. KLF4 is a transcription factor involved in cell cycle regulation, apoptosis, and differentiation. Its expression increases in response to DNA damage, serum deprivation, and contact inhibition [26]. Specifically, KLF4 has been shown to negatively regulate EMT in cancers. Down-regulation of KLF4 is required for EMT, cell migration, and for the induction of apoptosis [27]. Together, our results indicate that KLF4 may take part in the EMT progress induced by miR-10b in HCC.

Emerging evidence suggests that the Sp/KLF family member could regulate each other [28, 29]. We found that the transcriptional activity of KLF11 was reduced by KLF4 overexpression. The ChIP assays revealed that endogenous KLF4 directly bound to KLF11 promoter. Western-blot showed that KLF11 protein expression was downregulated or upregulated after overexpression or siRNA knockdown of KLF4, respectively. Altogether, KLF4 can bind to its special binding motifs on KLF11 promoter to down-regulate their transcription. Furthermore, the induction role of miR-10b in HCC EMT could be blocked by KLF11 siRNA. miR-10b promotes EMT in HCC cells via upregulation of KLF11.

## Conclusions

We first found that miR-10b could promote EMT in HCC cells. miR-10b downregulates KLF4, the inhibitory transcriptional factor of KLF11, which induces Smads signaling activity to promote HCC EMT. This newly identified miR-10b KLF4/KLF11/Smads pathway provides a new, potential therapeutic target to treat HCC.

## Abbreviations

HCC: hepatocellular carcinoma
EMT: epithelial–mesenchymal transition
miRNA: microRNA
ECM: extracellular matrix
MMP: matrix metalloproteinase
UTR: untranslated regions
KLF: Krüppel-like factor
RT-PCR: reverse transcription polymerase chain reaction
RNA: ribonucleic acid
mRNA: messenger RNA
GAPDH: glyceraldehyde-3-phosphate dehydrogenase
